# Precision cancer genome testing needs proficiency testing involving all stakeholders

**DOI:** 10.1038/s41598-022-05589-x

**Published:** 2022-01-27

**Authors:** Masato Maekawa, Terumi Taniguchi, Kazuto Nishio, Kazuko Sakai, Kazuyuki Matsushita, Kaname Nakatani, Takayuki Ishige, Makoto Ikejiri, Hiroshi Nishihara, Kuniko Sunami, Yasushi Yatabe, Kanako C. Hatanaka, Yutaka Hatanaka, Yoshihiro Yamamoto, Keita Fukuyama, Shinya Oda, Kayoko Saito, Mamoru Yokomura, Yuji Kubo, Hiroko Sato, Yoshinori Tanaka, Misa Fuchioka, Tadashi Yamasaki, Koichiro Matsuda, Kiyotaka Kurachi, Kazuhiro Funai, Satoshi Baba, Moriya Iwaizumi

**Affiliations:** 1 Department of Laboratory Medicine, Hamamatsu University School of Medicine, Hamamatsu, Japan; 2 Department of Genome Biology, Kindai University Faculty of Medicine, Sayama, Japan; 3 Department of Laboratory Medicine, Chiba University Hospital, Chiba, Japan; 4 Department of Clinical Laboratory, Mie University Hospital, Tsu, Japan; 5 Iga City General Hospital, Iga, Japan; 6 Genomics Unit, Keio Cancer Center, Keio University School of Medicine, Tokyo, Japan; 7 Department of Laboratory Medicine, National Cancer Center Hospital, Tokyo, Japan; 8 Department of Diagnostic Pathology, National Cancer Center Hospital, Tokyo, Japan; 9 Center for Development of Advanced Diagnostics, Hokkaido University Hospital, Sapporo, Japan; 10 Research Division of Genome Companion Diagnostics, Hokkaido University Hospital, Sapporo, Japan; 11 Department of Clinical Oncology, Kyoto University Hospital, Kyoto, Japan; 12 Cancer Genetics Laboratory, Clinical Research Institute, National Hospital Organization, Kyushu Cancer Center, Fukuoka, Japan; 13 Institute of Medical Genetics, Tokyo Women’s Medical Genetics, Tokyo, Japan; 14 Genetic Analysis Department, Tsukiji Registered Clinical Laboratory, Riken Genesis Co., Ltd., National Cancer Center, Tokyo, Japan; 15 Genetic Analysis Department, Kawasaki Registered Clinical Laboratory, RIKEN Genesis Co., Ltd., Life Innovation Center, Kawasaki, Japan; 16 Genetic & Pathology Department, SRL, Inc., Hachioji, Japan; 17 Development of Clinical Genomics, BML Inc., Kawagoe, Japan; 18 Molecular Genetic Analysis Department, Advanced Technology Center, LSI Medience Corporation, Tokyo, Japan; 19 Second Department of Surgery, Hamamatsu University School of Medicine, Hamamatsu, Japan; 20 First Department of Surgery, Hamamatsu University School of Medicine, Hamamatsu, Japan; 21 Department of Diagnostic Pathology, Hamamatsu University School of Medicine, Hamamatsu, Japan

**Keywords:** Cancer, Molecular medicine, Next-generation sequencing

## Abstract

To implement precision oncology, analytical validity as well as clinical validity and utility are important. However, proficiency testing (PT) to assess validity has not yet been systematically performed in Japan. To investigate the quality of next-generation sequencing (NGS) platforms and cancer genome testing prevalent in laboratories, we performed pilot PT using patient samples. We prepared genomic DNA from the cancer tissue and peripheral blood of 5 cancer patients and distributed these to 15 laboratories. Most participating laboratories successfully identified the pathogenic variants, except for two closely located *KRAS* variants and 25 bp delins in *EGFR*. Conversely, the *EGFR* L858R variant was successfully identified, and the allele frequency was similar for all the laboratories. A high DNA integrity number led to excellent depth and reliable NGS results. By conducting this pilot study using patient samples, we were able to obtain a glimpse of the current status of cancer genome testing at participating laboratories. To enhance domestic cancer genome testing, it is important to conduct local PT and to involve the parties concerned as organizers and participants.

## Introduction

The concept of personalized or precision medicine is expanding rapidly into practice and is based on cancer biology, including somatic variants, in particular driver variants. It is entirely dependent on the development of molecular methods, especially next-generation sequencing (NGS) technologies. NGS and cancer gene panels can simultaneously analyze multiple gene variants through efficient DNA sequencing and promote individualized treatment decision-making and precision medicine^1–3^. In contrast, molecular genetic testing using NGS is very complex because it comprises nucleic acid extraction, library preparation and sequencing chemistry, and bioinformatics pipelines. NGS technologies and databases are continuing to evolve, but the interpretation of NGS data remains challenging; therefore, optimization of the NGS process is required to obtain the correct results that lead to adequate treatment.

In Japan, cancer genome medicine has been promoted as a national strategy, and it is indispensable to ensure quality assurance for developing cancer genome testing. To implement precision oncology, analytical validity is as important as clinical validity and utility. Thus, to ensure suitable analytical validity, internal quality control and proficiency testing (PT) are essential. PT for cancer genome testing using NGS has been implemented in other countries using several types of testing specimens, primarily cell line-based samples and human genome DNA with spiked synthetic mutated DNA^1–10^. Yet, such PT has not been systematically performed in Japan. In this study, we have attempted to implement PT in patient samples to investigate the quality of NGS platforms and cancer genome testing usually used in laboratories. The purpose of this pilot PT is to ascertain the current quality status of cancer gene panel testing in Japan and disseminate the findings to stakeholders, including the participating laboratories, concerned academic societies, and policymakers.

## Results

### Variants reported in the pilot proficiency testing

The major variants reported by the laboratories and variant allele frequency (VAF) concordance with the coefficient of variation (CV) for five patient samples are shown in Supplementary Figs. 1, 2, 3, 5 and 6. Detailed information about the variants is shown in Supplementary Table 1, while clinically relevant variants detected in the five patient samples are summarized in Table 1. In two patient samples (nos. 1 and 5) clinically relevant variants were reported by all laboratories, but for the other patient samples (nos. 2, 3, and 4), they were not reported by some laboratories.

**Table 1:** Summary of PT results, including successfully reported clinically relevant variants.

Patient no.	Cancer location	Clinically relevant variants	No. of laboratories, reported/participated	Variant allele frequency		
				Mean (%)	Coefficient of variation (%)	ddPCR (%)
1	Rectum	*KRAS* p.Gly13Asp, NM_033360.2: c.38G>A	10/10, 100%	31	10.2	34
2	Rectum	*KRAS* p.Lys117Asn, c.351A>C	10/14, 71%	49	4.4	31
3	Lung	*EGFR* p.Thr751_Glu758del, NM_005228.3: c.2252_2275del ( 24 ) with c.2276T>A, or *EGFR* p.Thr751_Ile759delinsN c.2252_2276delinsA	5/11, 45%	26	47.1	Not analyzed
4	Colon	*BRAF* p.Val600Glu, NM_004333.4: c.1799T>A	8/10, 80%	21	41.8	20
5	Lung	*EGFR* p.Leu858Arg, c.2573T>G	15/15, 100%	60	2.4	61

The sample from Patient No. 1 was analyzed by ten laboratories, and all of them reported the *KRAS* p.Gly13Asp (NM_033360.2: c.38G>A) missense variant with similar VAF to the droplet digital PCR (ddPCR) result. *KRAS* is an actionable and druggable gene, and detection of its pathological variants will lead to a precise therapeutic strategy.

The sample from Patient No. 2 was analyzed by 14 laboratories, and 10 laboratories reported the *KRAS* p.Lys117Asn (c.351A>C) variant, with two laboratories using the Oncomine Dx target test. The allele frequencies of the variants were almost similar. Another *KRAS* p.Leu120Met (c.358T>A) variant was reported by seven laboratories (Supplementary Fig. 2). One laboratory detected the variants but did not report any clinical utility, and four laboratories could not detect the variant due to filtering out in the bioinformatics pipeline.

The sample from Patient No. 3 was analyzed by 11 laboratories. Five laboratories reported the deletion variants in *EGFR* exon 19 (Supplementary Fig. 3); however, the deletion start site was different, resulting in additional amino-acid substitutions. In exon 19 of *EGFR*, many types of deletion variants have been discovered; there are several registries about these similar variants in COSMIC, as shown in Table 2. We performed Sanger sequencing and concluded that this patient had a complex variant of *EGFR* exon 19 (Supplementary Fig. 4). The correct variant identification was a combination of NM_005228.3: c.2252_2275del (COSMIC ID 23634) and c.2276T>A (I759N; ID 23633), or c.2252_2276delinsA (ID 96856), and was reported by two laboratories. Three laboratories reported the wrong deletion start site, c.2253_2276del (ID 13556). The difference was only one nucleotide from the start site of the 24 bp deletion. One laboratory reported the I759N (c.2276T>A) missense variant only. Other laboratories reported no variants in *EGFR*. That is, 6 of the 11 laboratories detected variants in exon 19 of *EGFR*, leading to a precise therapeutic strategy. Two in vitro diagnostics (IVD) reagents for companion diagnostics, the Therascreen *EGFR* mutation detection kit RGQ and Cobas *EGFR* Mutation Test v2, along with the Oncomine Dx target test, were designed to be able to identify some types of deletion variants in exon 19; however, they could not identify the deletion variants in the patient 3.

**Table 2:**
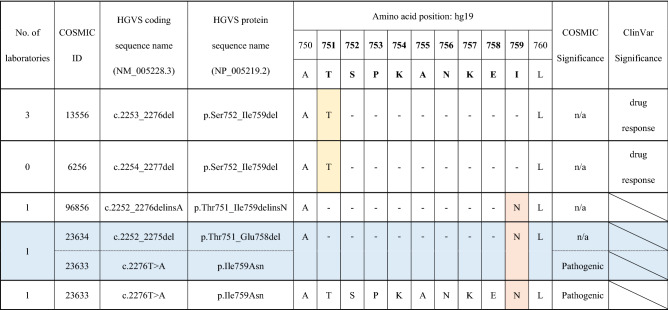
Deletion variant in *EGFR* exon 19 in Patient 3.

The variant results obtained from NGS and Sanger sequencing (Supplementary Fig. 4) are summarized. Five types of variants were reported. Deletion variants identified by laboratories were slightly different. However, the correct variant identification is COSMIC ID 96856 or ID 23634 + 232633, which causes deletion of 8 amino acids and a Thr to Asn amino acid substitution, resulting in a possibly pathogenic variant.

HGVS: Human Genome Variation Society.

The sample from Patient No. 4 was analyzed by 10 laboratories, and 8 laboratories reported the *BRAF* V600E variant (NM_004333.4: c.1799T>A). Two laboratories using the Oncomine Dx target test did not report this variant, but variant reads were found in the Integrative Genomics Viewer (IGV) files. Most laboratories successfully detected and reported this variant; however, the allele frequency of this variant varied from 8 to 39% in laboratories using the amplicon-based procedure. Moreover, ddPCR indicated that the allele frequency was approximately 20%, which was close to the mean of the results from the reported laboratories. Many other variants have been identified and reported (Supplementary Fig. 5). This is probably a result of microsatellite instability (MSI) due to the dysfunction of the mismatch repair genes.

The sample from Patient No. 5 was analyzed by 15 laboratories, and all of them reported the *EGFR* L858R variant (NM_005228.3: c.2573T>G), leading to a precise therapeutic strategy. The allele frequencies of the variant in the laboratories converged in a narrow range and was very similar to the value obtained from ddPCR (Table 1, Supplementary Fig. 6).

Germline variants were detected and reported in patients 1, 3, and 5 in a few laboratories (Supplementary Figs. 1, 3 and 6). The variants were *BRCA1* (NM_007300.3: c.3022A>G), *STK11* (NM_000455.4: c.842C>T) and *Ret* (NM_020975.4: c.1946C>T), however, these were not clinically relevant and were not necessarily reported by all laboratories.

### Relation between DNA quality and NGS result

**Figure 1:**
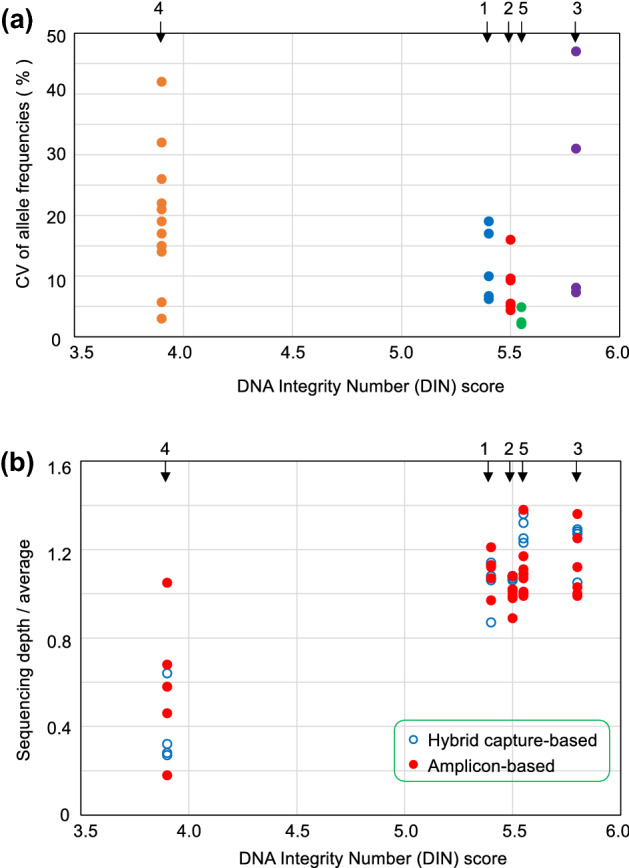
Correlation between DNA quality and NGS results. (**a**) Correlation between DNA quality and coefficient of variation (CV) of allele frequencies for each mutation. Arrows and numbers indicate the patient number. Each circle indicates the CV of allele frequencies of the detected variants. (**b**) Correlation between DNA quality and depth of sequencing coverage for each laboratory. Arrows and numbers indicate the patient number. Open and closed circles indicate the capture-based and amplicon-based methods, respectively.

We considered the effect of DNA quality on NGS results. Figure 1a shows the correlation between the DNA quality and CV of allele frequencies. DNA prepared from formalin-fixed paraffin-embedded (FFPE) with lower tumor density showed lower VAF and higher CV% (Patient No. 4, Supplementary Fig. 5); VAF tended to be inversely correlated with the CV% of allele frequencies. Patient No. 3 presented a high CV of allele frequencies because of the 24 bp deletion and an adjacent single nucleotide variant, which seem to be unique. The ratio of sequencing depth against the DNA Integrity Number (DIN) of each sample among the laboratories is shown in Fig. 1b. A high DIN leads to excellent depth and possibly secure and reliable NGS results. The library preparation procedure was not considered as an influencing factor. The DNA samples from Patient No. 4 had lower DIN scores with significantly higher CV% of allele frequencies (*p* = 0.032) and lower ratio of sequencing depth/average (*p* = 0.00012) than the DNA samples from the other patients by the Student’s *t*-test (Fig. 1a,b). This implies that low DIN scores affect the quality of NGS results and might be descriptive of the importance of preanalytical processes, especially DNA preparation.

## Discussion

In this study, we performed a pilot PT study to share the current quality status of cancer gene panel testing in Japan with stakeholders. PT samples are usually derived from cell line-based samples^7^ or mixtures of the human genome and synthetic DNA-based samples^5,9^. Cell lines have the advantage that they are prepared with known ratios to simulate different VAFs; however, they might be expensive and time-consuming, or have sequence artifacts due to genetic manipulation and non-physiological patterns^4,6^. Synthetic DNA fragments have the advantage that they can be designed to harbor a broad range of sequence variants and can be used as spike-in standards; however, they may have limited length, resulting in artifacts being detected by NGS platforms^4,6^. The ideal PT samples should be obtained from clinical specimens and should allow all phases of the testing process to be evaluated. Yet, archival FFPE tissue specimens are not easily available for large-scale PT studies because of the limited number of tumor and normal tissue samples available from a given patient^5^.

Our concept focuses on the ACCE model (analytic validity, clinical validity, clinical utility, and ethical, legal and social implications) for evaluating genetic tests using CDC (Centers for Disease Control and Prevention)^11^. The NGS technology consists of preanalytical, analytical, and post-analytical processes, similar to any other specimen examination. Some bioinformatics pipelines are designed for matched pair analysis using cancer and normal DNA. Therefore, we distributed purified DNA for PT samples to compare only NGS analysis findings and to avoid differences among laboratories with respect to DNA purification quality and the portion of FFPE. From this, all types of NGS workflows could be adequately evaluated using a pair of DNA samples from cancer and normal tissues.

We prepared both cancer and normal DNA samples in this PT study, and a few laboratories reported DNA germline variants in patients 1, 3, and 5. Because possible relevant variants causing familial cancer or hereditary disorders were not detected in the five patients, we did not consider genetic counseling for germline variants or hereditary disorders.

In the sample from Patient No. 2, two variants in *KRAS* were confirmed to be located on the same allele by visual inspection of the IGV file, and identified by Sanger sequencing. The two variants were very close on the same allele and could not be easily identified using bioinformatics. Once the variant was removed, we could not confirm the IGV file visually, resulting in false negatives. This result seems to be an issue of bioinformatics programs. Multiple variants at a close position might be programmed to be filtered out because of unreliable sequencing. Deletion variants like the sample from Patient No. 3 might be also likely misidentified by bioinformatics.

The detection failure of the relevant *BRAF* variant and variation in allele frequency in the sample of Patient No. 4 might be explained by the following: the tumor density was the lowest (25%), the FFPE of this tumor tissue was the oldest among the five testing samples, and the DIN was the lowest (3.9). The dispersed VAF might cause this variant to be overlooked, especially around the detection limit; therefore, in such cases, we should consider using PCR amplification for library preparation and a bioinformatics detection algorithm in particular. Because this patient had so many variants, MSI was investigated. All 5 mononucleotide repeat markers and one pentanucleotide repeat marker showed additional bands and were classified as microsatellite instability-high (MSI-H) (Supplementary Fig. 7). Immunohistochemistry analysis demonstrated positive staining of MSH2 and MSH6 and negative staining of MLH1 and PMS2 (Supplementary Fig. 8). These results are highly suggestive of reduced MLH1 expression.

An important aspect of this patient is the possibility of detecting the *BRAF* V600E mutation. The *BRAF*-V600E variant has been well studied in many neoplasms, most commonly in melanoma, and for which targeted therapies are available^12^. In colorectal cancer, *BRAF*-V600E targeted therapies have been less successful, but *BRAF*-mutant metastatic colorectal cancer is associated with a worse prognosis. Therefore, timely ascertainment of molecular subtypes is critically important for treatment planning and earlier consideration of targeted therapies^13^.

Generally, the major variants tended to be adequately reported; however, certain variants could not be precisely detected in some laboratories. Most laboratories specified the variants with clinical utility, but in some samples where multiple variants were closely located or had complex deletion variants, they were more difficult to detect. These detection failures may be primarily caused by the bioinformatics pipeline. Reportable ranges were different, and variants reported because of their clinical significance were also different in each laboratory. Conflicting interpretations of pathogenicity and variants of uncertain significance should be reduced with the improvement of variant databases. Moreover, an easily understandable, useful, and precise report format by molecular pathologists, laboratory physicians, medical scientists, and bioinformaticians is necessary, and standardization of the interpretation and reporting in cancer genomic medicine should be considered^14^.

We recognize the strengths and weaknesses of our NGS analysis as this PT study was an extremely valuable educational opportunity. One strength of this pilot PT is knowledge on the current quality of any NGS platforms and cancer genome testing usually used in laboratories. However, there are some limitations associated with our study: the correct answers for each specimen were not perfectly decided; the variants detected in these randomly selected patients may not necessarily be represented in clinical settings; and the number of the participating laboratories is small.

We learned something valuable in conducting the first PT of cancer gene panels in Japan. In many countries with advanced cancer genome testing, such PTs are undertaken by their own governments, parties, or national organizations, and many laboratories participate in the PT, resulting in giving back detailed information to all participants and stakeholders. However, in countries like Japan with underdeveloped cancer genome testing, such PT has not been planned and hosted. To advance cancer genome testing and precision medicine, a permanent institution for quality assurance is recommended, and our own PT shall be periodically built, developed and orchestrated by all stakeholders including academia, industries, with the financial support and cooperation of policymakers.

## Conclusion

By conducting this pilot study using patient samples, we were able to obtain a glimpse of the current status of cancer genome testing at participating laboratories, and undertaking the PT locally was valuable for information sharing. To enhance cancer genome testing, it is important to conduct PT locally and to involve the parties concerned as organizers and participants.

## Materials and methods

### Samples for PT

Patient samples, obtained from the Department of Diagnostic Pathology at Hamamatsu University Hospital, comprised FFPE tissues from five patients with lung or colorectal cancer; matched normal samples were obtained from peripheral blood. These patients were selected based on their relatively large tumor masses and on providing informed consent. The clinical and pathological features of the patients are presented in Supplementary Table 2. The histological findings were also supplied to the participating laboratories.

The genomic DNA (gDNA) was prepared using the QIAamp DNA FFPE Tissue Kit and Deparaffinization Solution (QIAGEN, Hilden, Germany) from FFPE and the EZ1 DNA Blood Kit (QIAGEN) from the whole blood. The DNA quantity and quality were measured using a 2200 TapeStation (Agilent Technologies, Santa Clara, CA) system using the TapeStation Analysis software (Agilent), which automatically determines and displays the DIN. The properties of the DNA samples are presented in Table 3.

**Table 3:** Properties of the patient samples.

	Patient ID	Origin	Tumor density	Cancer location	DNA quantity(ng/µL)	Distributed sample volume (µL)	Optical density ratio (260 nm/280 nm)	DIN
Essential examination	2 T	FFPE	60%	Rectum	241.0	10	2.02	5.5
	2 N	Blood			85.4	10	1.87	9.8
	5 T	FFPE	60%	Lung	209.0	10	1.98	5.5
	5 N	Blood			78.8	10	1.79	9.5
Optional examination	1 T	FFPE	50%	Rectum	380.0	10	2.01	5.4
	1 N	Blood			79.0	10	1.80	9.7
	3 T	FFPE	65%	Lung	172.0	10	1.97	5.8
	3 N	Blood			56.7	10	1.82	9.3
	4 T	FFPE	25%	Colon	168.0	10	1.90	3.9
	4 N	Blood			108.0	10	1.83	9.1

This study was approved by the Institutional Review Board of Hamamatsu University School of Medicine (19-186). Five pairs of FFPE and peripheral blood samples were prepared with the patients’ written informed consent. All methods were carried out in accordance with the Declaration of Helsinki.

### PT participants

Fifteen clinical laboratories (12 hospital clinical laboratories and 3 registered clinical laboratories) participated in this study. The sequencing assay examined in this study was independently validated and implemented in each laboratory. Five laboratories used the hybrid capture-based method, and 10 laboratories used the amplicon-based method for NGS library preparation. Seven laboratories analyzed paired samples of tumor (T) tissues and normal blood cells (N), and eight used tumor tissues only. Supplementary Table 3 provides a brief explanation of the laboratories' analytic procedures. The analysis platform, gene panel (targeted genes, all coding exons or hotspot only), bioinformatics pipeline, and reportable range were different among the laboratories.

We tentatively decided that two samples (colorectal cancer from Patient No. 2 and lung cancer from Patient No. 5) were essential for all laboratories, and the remaining three samples (patient nos. 1, 3, and 4) were optional. Each participating laboratory selected either the two essential samples, all five samples, or another set of samples based on their NGS platform or financial capacities. The number of analyzed sample in each laboratory are indicated in Supplementary Table 3. PT samples were sent to the participating laboratories on March 24, 2020, with information about the DNA quality and quantity of the samples and the patients’ pathological and clinical findings.

We collected the analysis reports from all participating laboratories at the end of May 2020, and then asked whether the consensus mutations could be detected by NGS analysis and the reason in case they were not reported, including lack of clinical utility.

### Additional analysis method for the PT samples

The PT specimens were also analyzed using in vitro diagnostics (IVD) reagents in a commercial laboratory accredited by ISO 15189 and CLIA. For lung cancer, the *EGFR* variants were analyzed using the Therascreen *EGFR* mutation detection kit RGQ based on Scorpion-ARMS (QIAGEN) and Cobas *EGFR* Mutation Test v2 (Roche Diagnostics, Basel, Switzerland) based on a real-time PCR test. For colorectal cancer, the *RAS* and *BRAF* variants were examined using the MEBGEN RASKET-B kit based on PCR-reverse sequence-specific oligonucleotide (PCR-rSSO) (MBL, Nagoya, Japan) and a Cobas *BRAF* V600 detection kit based on real-time PCR (Roche Diagnostics).

The allele frequencies of the major variants were quantified using a QX200 AutoDG Droplet Digital PCR system (Bio-Rad Laboratories Inc., Hercules, CA, USA), and the data were analyzed using the Bio-Rad QuantaSoft Analysis Pro. All ddPCR experiments were performed in triplicate.

Sanger sequencing was performed using the BigDye Terminator v3.1 cycle sequencing Kit (ABI, Foster City, CA) and the ABI 3500xL Genetic Analyzer (ABI). Microsatellite instability (MSI) was detected by a Promega MSI Analysis System with five mononucleotide repeat markers (BAT-26, BAT-25, NR-21, NR-24 and MONO-27) according to the manufacturer's protocol (Promega, Madison, WI). Samples with ≥ 2 altered markers out of 5 were classified as MSI-H. Two pentanucleotide repeat markers (Penta-C and Penta-D) were used to confirm that the test sample and the paired normal samples were from the same individual.

Immunohistochemistry staining for the expression of 4 mismatch repair proteins (MLH1, MSH2, PMS2 and MSH6) was performed as described previously^15^.

### Statistical analysis

Statistical analyses were performed using Microsoft Excel 2017 for Windows, and *P* < 0.05 was considered statistically significant.

### Ethics declarations

This study was approved by the Institutional Review Board of the Hamamatsu University School of Medicine (19-186) according to the ethical guidelines of the Japanese government. Written informed consent was obtained from the individuals for obtaining samples of FFPE and peripheral blood. All methods were carried out in accordance with the Declaration of Helsinki.

## Supplementary Information

Supplementary Information.
