# Supplementary material for: Precision cancer genome testing needs proficiency testing involving all stakeholders
Source: Sci Rep. 2022 Jan 27;12:1494. doi: 10.1038/s41598-022-05589-x (PMC8795413; doi:10.1038/s41598-022-05589-x)
Supplement: Supplementary file 1 — Supplementary Information. [file 41598_2022_5589_MOESM1_ESM.pdf]

**Precision cancer genome testing needs proficiency testing involving all stakeholders**

Masato Maekawa, Terumi Taniguchi, Kazuto Nishio, Kazuko Sakai, Kazuyuki Matsushita, Kaname Nakatani, Takayuki Ishige, Makoto Ikejiri, Hiroshi Nishihara, Kuniko Sunami, Yasushi Yatabe, Kanako C Hatanaka, Yutaka Hatanaka, Yoshihiro Yamamoto, Keita Fukuyama, Shinya Oda, Kayoko Saito, Mamoru Yokomura, Yuji Kubo, Hiroko Sato, Yoshinori Tanaka, Misa Fuchioka, Tadashi Yamasaki, Koichiro Matsuda, Kiyotaka Kurachi, Kazuhiro Funai, Satoshi Baba, Moriya Iwaizumi

**Supplementary Information (Supplementary Figure Legends)**

**Supplementary Figure 1. Report summary for Patient 1**

**a)** Variants reported by the participating laboratories. The *KRAS* p.Gly13Asp (NM_033360.2: c.38G>A) missense variant highlighted in red was confirmed using the MEBGEN RASKET-B kit (PCR-rSSO). ddPCR analysis indicated that the allele frequency was approximately 34%, which was quite similar to the results in the NGS analysis. (G) represents the germline variant. The *BRCA1* missense germline variant, p.Met1008Val (NM_007300.3: c.3022A>G), was reported by four laboratories using the N/T sample pairs and one laboratory using T sample only. This germline variant is possibly not pathogenic. The VAFs reported by the six laboratories were distributed at around 50%.

**b)** Report status for the variants indicated in a). “1st time” indicates initial reporting by the laboratories, “2nd time” indicates reporting after follow up regarding the existence of the variant, and “not detected” indicates no reporting after the follow up.

**c)** Variant allele frequency (VAF) of the reported variants. Blue diamonds and red circles indicate capture-based and amplicon-based methods, respectively. The green vertical bar indicates the ddPCR results, assayed in triplicate.

**d)** Coefficient of Variation (CV) (%) of the VAFs in the laboratory reports.

**Supplementary Figure 2. Report summary for Patient 2**

**a)** Variants reported by the participating laboratories. The *KRAS* p.Lys117Asn (c.351A>C) variant highlighted in red was revealed using RASKET-B kit. The *KRAS* p.Leu120Met (c.358T>A) variant was not included in the reportable range of the RASKET-B kit and Oncomine Dx target test. Both of these *KRAS* variants were confirmed using Sanger sequencing.

**b)** Report status for the variants indicated in a). "1st time" indicates initial reporting by the laboratories, "2nd time" indicates reporting after follow up regarding the existence of the variant, and "not detected" indicates no reporting after the follow up.

**c)** Variant Allele Frequency (VAF) of the reported variants. Blue diamonds and red circles indicate capture-based and amplicon-based methods, respectively. The green vertical bar indicates the ddPCR results, assayed in triplicate.

**d)** Coefficient of variation (CV) (%) of the VAFs in the laboratory reports.

**Supplementary Figure 3. Report summary for Patient 3.**

**a)** Variants reported by the participating laboratories. The variants highlighted in red are detected because of their relationship with therapeutics. (G) represents the germline variant (*STK11* p.Pro281Leu, NM_000455.4: c.842C>T) which was reported by seven laboratories; however, the variant allele frequencies (VAFs) from four laboratories were slightly lower than the theoretical 50%.

**b)** Report status for the variants indicated in a). "1st time" indicates initial reporting by the laboratories, "2nd time" indicates reporting after follow up regarding the existence of the variant, and "not detected" indicates no reporting after the follow up.

**c)** VAF of the reported variants. Blue diamonds and red circles indicate capture-based and amplicon-based methods, respectively.

**d)** Coefficient of variation (CV) (%) of the VAFs in the laboratory reports.

**Supplementary Figure 4. Sanger sequencing of *EGFR* exon 19 in Patient 3**

Genomic DNA was amplified by PCR using primers for the variant region and was directly sequenced by both forward and reverse sequencing. A characteristic overlapped pattern was observed, indicating a frameshift, and the starting point of the frameshift was identified. 3T and 3N indicate tumor cells and normal cells (blood cells) of Patient 3, respectively.

**Supplementary Figure 5. Report summary for Patient 4.**

**a)** Variants reported by the participating laboratories. The variant highlighted in red are detected because of their relationship with therapeutics.

**b)** Report status for the variants indicated in a). “1st time” indicates initial reporting by the laboratories, “2nd time” indicates reporting after follow up regarding the existence of the variant, and “not detected” indicates no reporting after the follow up.

**c)** Variant allele frequency (VAF) of the reported variants. Blue diamonds and red circles indicate capture-based and amplicon-based methods, respectively. The green vertical bar indicates the ddPCR results, assayed in triplicate.

**d)** Coefficient of variation (CV) (%) of the VAFs in the laboratory reports.

**Supplementary Figure 6. Report summary for Patient 5.**

**a)** Variants reported by the participating laboratories. The *EGFR* L858R (NM_005228.3: c.2573T>G) variant highlighted in red should be detected because of its relationship with therapeutics. This variant was reported by two *in vitro* diagnostic (IVD) reagents, the Therascreen *EGFR* mutation detection kit RGQ and Cobas *EGFR* Mutation Test v2. The *EGFR* A289V mutation was reported by all participating laboratories with similar allele frequencies. However, two IVD reagents did not report this variant,

probably because the variant was not within their reportable range. Twelve laboratories reported the *RET* germline variant represented by (G) (p.Ser649Leu, NM_020975.4: c.1946C>T), but three laboratories using the Oncomine Dx target test kit failed to report beyond the reportable range. This variant is positioned in a conflicting interpretation of pathogenicity by ClinVar.

**b)** Report status for the variants indicated in a). "1st time" indicates initial reporting by the laboratories, "2nd time" indicates reporting after follow up regarding the existence of the variant, and "not detected" indicates no reporting after the follow up.

**c)** Variant allele frequency (VAF) of the reported variants is presented. Blue diamonds and red circles indicate capture-based and amplicon-based methods, respectively. The vertical bar indicates the ddPCR results, assayed in triplicate.

**d)** Coefficient of variation (CV) (%) of the VAFs in the laboratory reports.

**Supplementary Figure 7. Microsatellite instability analysis of Patient 4**

Microsatellite instability analysis using five mononucleotide repeat markers (BAT-26, BAT-25, NR-21, NR-24 and MONO-27). All five markers and one pentanucleotide marker (Penta-C) in cancer tissue showed additional peaks (shown by red arrows) compared with the results from blood samples, and microsatellite instability-high (MSI-H) was detected in Patient 4.

**Supplementary Figure 8. Immunohistochemistry analysis of Patient 4**

Immunohistochemistry staining for the expression of 4 mismatch repair proteins (MLH1, MSH2, PMS2 and MSH6) was performed, and negative staining of MLH1 and PMS2 was observed.

**Supplementary Figure 2. Report summary for Patient 2**

**a)** Variants reported by the participating laboratories. The *KRAS* p.Lys117Asn (c.351A>C) variant highlighted in red was revealed using RASKET-B kit. The *KRAS* p.Leu120Met (c.358T>A) variant was not included in the reportable range of the RASKET-B kit and Oncomine Dx target test. Both of these *KRAS* variants were confirmed using Sanger sequencing.

**b)** Report status for the variants indicated in a). “1st time” indicates initial reporting by the laboratories, “2nd time” indicates reporting after follow up regarding the existence of the variant, and “not detected” indicates no reporting after the follow up.

**c)** Variant Allele Frequency (VAF) of the reported variants. Blue diamonds and red circles indicate capture-based and amplicon-based methods, respectively. The green vertical bar indicates the ddPCR results, assayed in triplicate.

**d)** Coefficient of variation (CV) (%) of the VAFs in the laboratory reports.

**Supplementary Figure 3. Report summary for Patient 3.**

**a)** Variants reported by the participating laboratories. The variants highlighted in red are detected because of their relationship with therapeutics. (G) represents the germline variant (*STK11* p.Pro281Leu, NM_000455.4: c.842C>T) which was reported by seven laboratories; however, the variant allele frequencies (VAFs) from four laboratories were slightly lower than the theoretical 50%.

**b)** Report status for the variants indicated in a). “1st time” indicates initial reporting by the laboratories, “2nd time” indicates reporting after follow up regarding the existence of the variant, and “not detected” indicates no reporting after the follow up.

**c)** VAF of the reported variants. Blue diamonds and red circles indicate capture-based and amplicon-based methods, respectively.

**d)** Coefficient of variation (CV) (%) of the VAFs in the laboratory reports.

**Supplementary Figure 4. Sanger sequencing of *EGFR* exon 19 in Patient 3**

Genomic DNA was amplified by PCR using primers for the variant region and was directly sequenced by both forward and reverse sequencing. A characteristic overlapped pattern was observed, indicating a frameshift, and the starting point of the frameshift was identified. 3T and 3N indicate tumor cells and normal cells (blood cells) of Patient 3, respectively.

**Supplementary Figure 5. Report summary for Patient 4.**

**a)** Variants reported by the participating laboratories. The variant highlighted in red are detected because of their relationship with therapeutics.

**b)** Report status for the variants indicated in a). “1st time” indicates initial reporting by the laboratories, “2nd time” indicates reporting after follow up regarding the existence of the variant, and “not detected” indicates no reporting after the follow up.

**c)** Variant allele frequency (VAF) of the reported variants. Blue diamonds and red circles indicate capture-based and amplicon-based methods, respectively. The green vertical bar indicates the ddPCR results, assayed in triplicate.

**d)** Coefficient of variation (CV) (%) of the VAFs in the laboratory reports.

**Supplementary Figure 6. Report summary for Patient 5.**

**a)** Variants reported by the participating laboratories. The *EGFR* L858R (NM_005228.3: c.2573T>G) variant highlighted in red should be detected because of its relationship with therapeutics. This variant was reported by two *in vitro* diagnostic (IVD) reagents, the Therascreen *EGFR* mutation detection kit RGQ and Cobas *EGFR* Mutation Test v2. The *EGFR* A289V mutation was reported by all participating laboratories with similar allele frequencies. However, two IVD reagents did not report this variant,

probably because the variant was not within their reportable range. Twelve laboratories reported the *RET* germline variant represented by (G) (p.Ser649Leu, NM_020975.4: c.1946C>T), but three laboratories using the Oncomine Dx target test kit failed to report beyond the reportable range. This variant is positioned in a conflicting interpretation of pathogenicity by ClinVar.

**b)** Report status for the variants indicated in a). “1st time” indicates initial reporting by the laboratories, “2nd time” indicates reporting after follow up regarding the existence of the variant, and “not detected” indicates no reporting after the follow up.

**c)** Variant allele frequency (VAF) of the reported variants is presented. Blue diamonds and red circles indicate capture-based and amplicon-based methods, respectively. The vertical bar indicates the ddPCR results, assayed in triplicate.

**d)** Coefficient of variation (CV) (%) of the VAFs in the laboratory reports.

**Supplementary Figure 7. Microsatellite instability analysis of Patient 4**

Microsatellite instability analysis using five mononucleotide repeat markers (BAT-26, BAT-25, NR-21, NR-24 and MONO-27). All five markers and one pentanucleotide marker (Penta-C) in cancer tissue showed additional peaks (shown by red arrows) compared with the results from blood samples, and microsatellite instability-high (MSI-H) was detected in Patient 4.

**Supplementary Figure 8. Immunohistochemistry analysis of Patient 4**

Immunohistochemistry staining for the expression of 4 mismatch repair proteins (MLH1, MSH2, PMS2 and MSH6) was performed, and negative staining of MLH1 and PMS2 was observed.

**Patient 1**
Rectum cancer
Tumor density=50%

(a)

*KRAS* **p.Gly13Asp c.38G>A**

*APC* p.Trp421Ter c.1263G>A

*APC* p.Asp1318ValfsTer5 c.3948_3952dup

*PDGFRA* p.Arg804Gln c.2411G>A

*CTNNB1* p.Arg565Cys c.1693C>T

*BRCA1* (G) p.Met1008Val c.3022A>G

(b)

| No. of laboratories | | | |
|---|---|---|---|
| Report | | | out of reportable range |
| 1st time | 2nd time | not detected | |
| 10 | 0 | 0 | 0 |
| 4 | 0 | 1 | 5 |
| 6 | 0 | 0 | 4 |
| 5 | 1 | 0 | 4 |
| 5 | 0 | 0 | 5 |
| 5 | 1 | 0 | 4 |

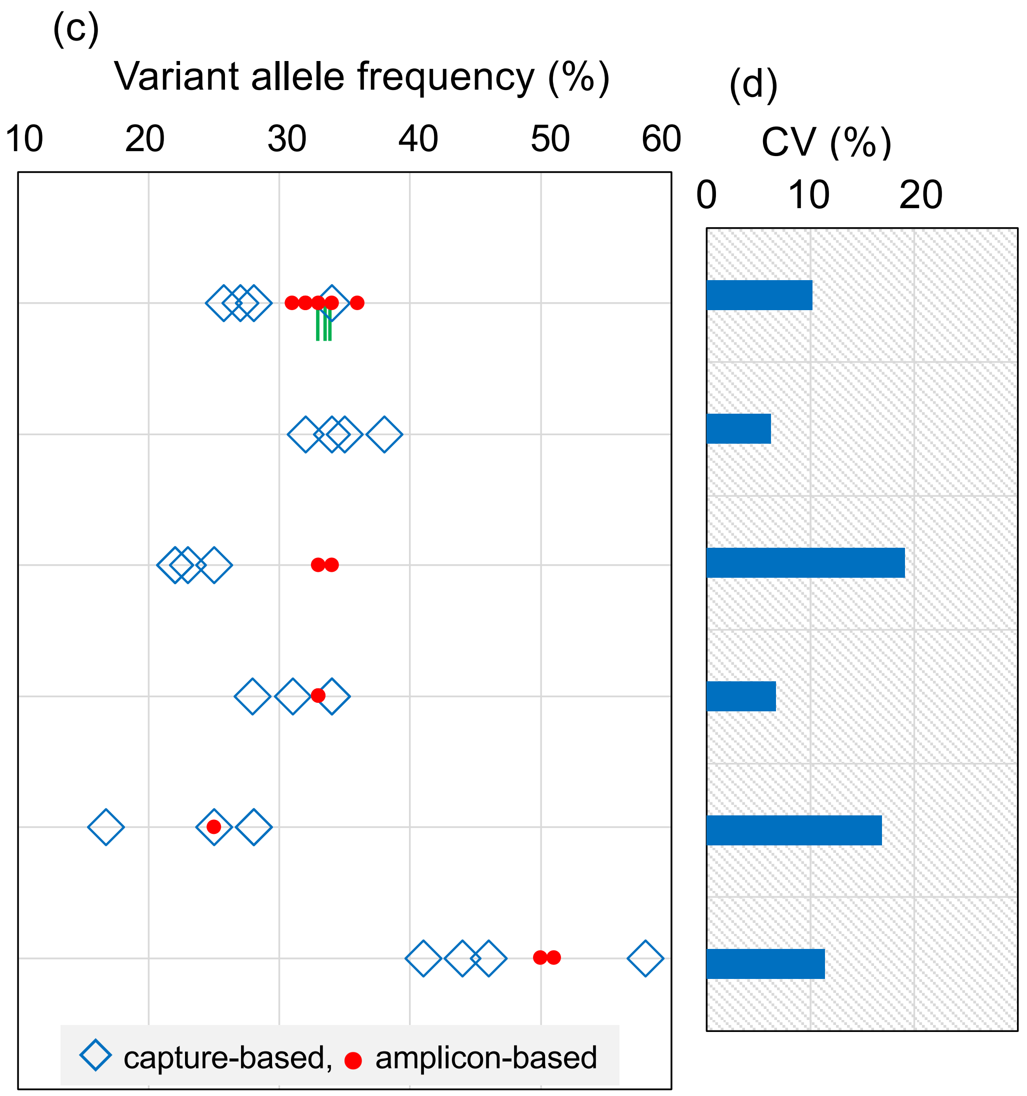


Supplementary Fig. 1

**Patient 2**
Rectum cancer
Tumor density=60%

(a)

| Variant |
|---|
| **_KRAS_ p.Lys117Asn c.351A>C** |
| _KRAS_ p.Leu120Met c.358T>A |
| _APC_ p.Trp1049Ter c.3147G>A |
| _APC_ p.Glu1309AspfsTer4 c.3927_3931del |
| _MYC_ p.Tyr417LeufsTer15 c.1249dup |
| _CTNNB1_ p.Lys270Thr c.809A>C |

(b)

| No. of laboratories | | | |
|---|---|---|---|
| Report | | | out of reportable range |
| 1st time | 2nd time | not detected | |
| 10 | 3 | 1 | 0 |
| 7 | 5 | 0 | 2 |
| 8 | 0 | 0 | 6 |
| 9 | 0 | 0 | 5 |
| 5 | 2 | 1 | 6 |
| 7 | 0 | 1 | 6 |

(c)

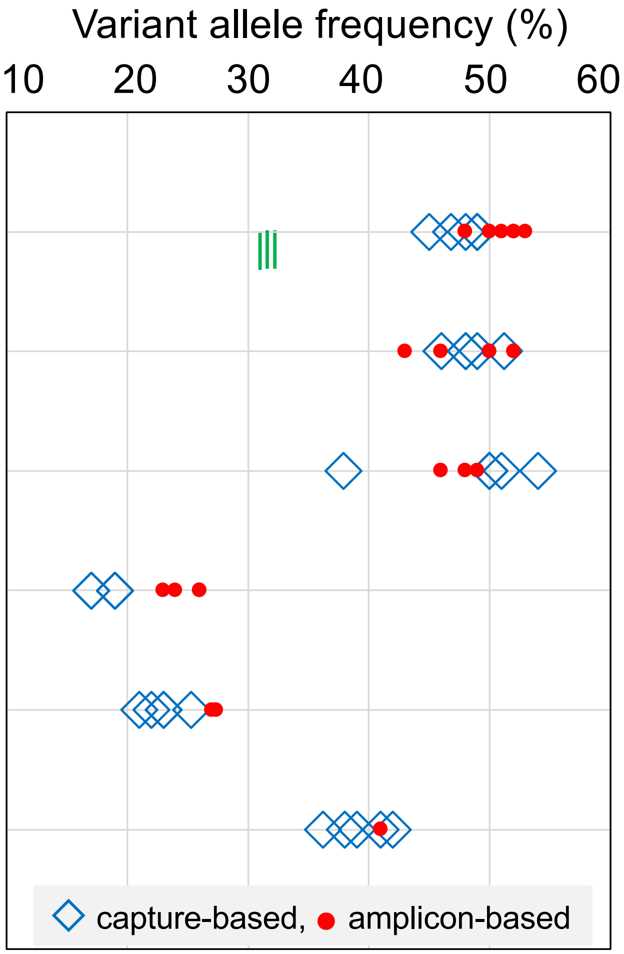


(d)

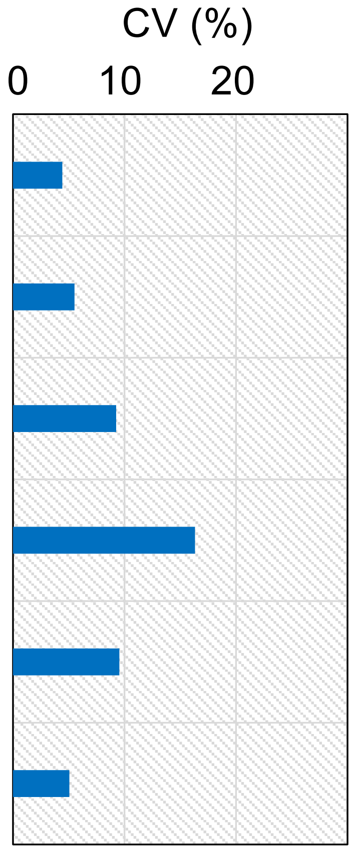


Supplementary Fig. 2

(a)

**Patient 3**
Lung cancer
Tumor density=65%

| Variant | (b) No. of laboratories: Report 1st time | Report 2nd time | Report not detected | out of reportable range |
|---|---|---|---|---|
| ***EGFR* p.Thr751_Glu758del c.2252_2275del ( 24 )** | 5 | 0 | 3 | 3 |
| ***EGFR* p.Ile759Asn c.2276T>A** | 3 | 0 | 5 | 3 |
| *TP53* p.Pro80HisfsTer68 c.239_240del | 6 | 0 | 1 | 4 |
| *FGFR3* p.Met433Val c.1297A>G | 4 | 1 | 0 | 6 |
| *STK11* *(G)* p.Pro281Leu c.842C>T | 3 | 4 | 0 | 4 |

(c)

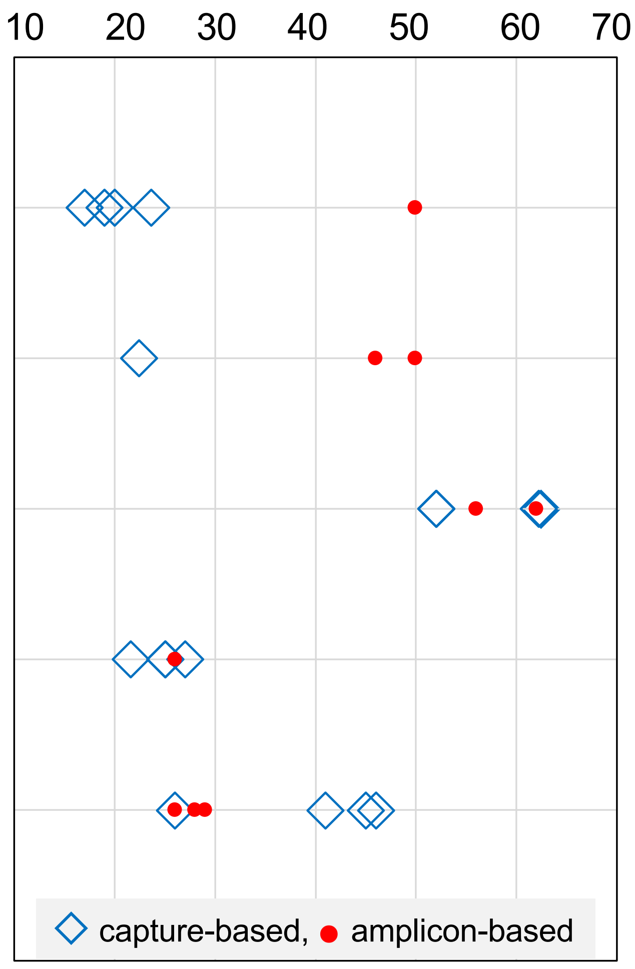


(d)

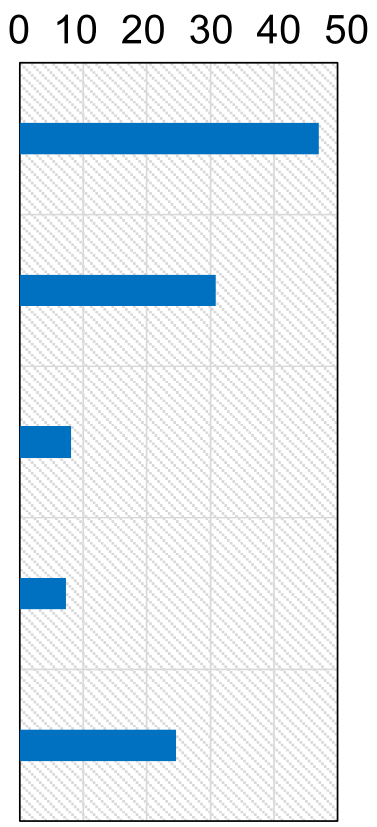


Supplementary Fig. 3

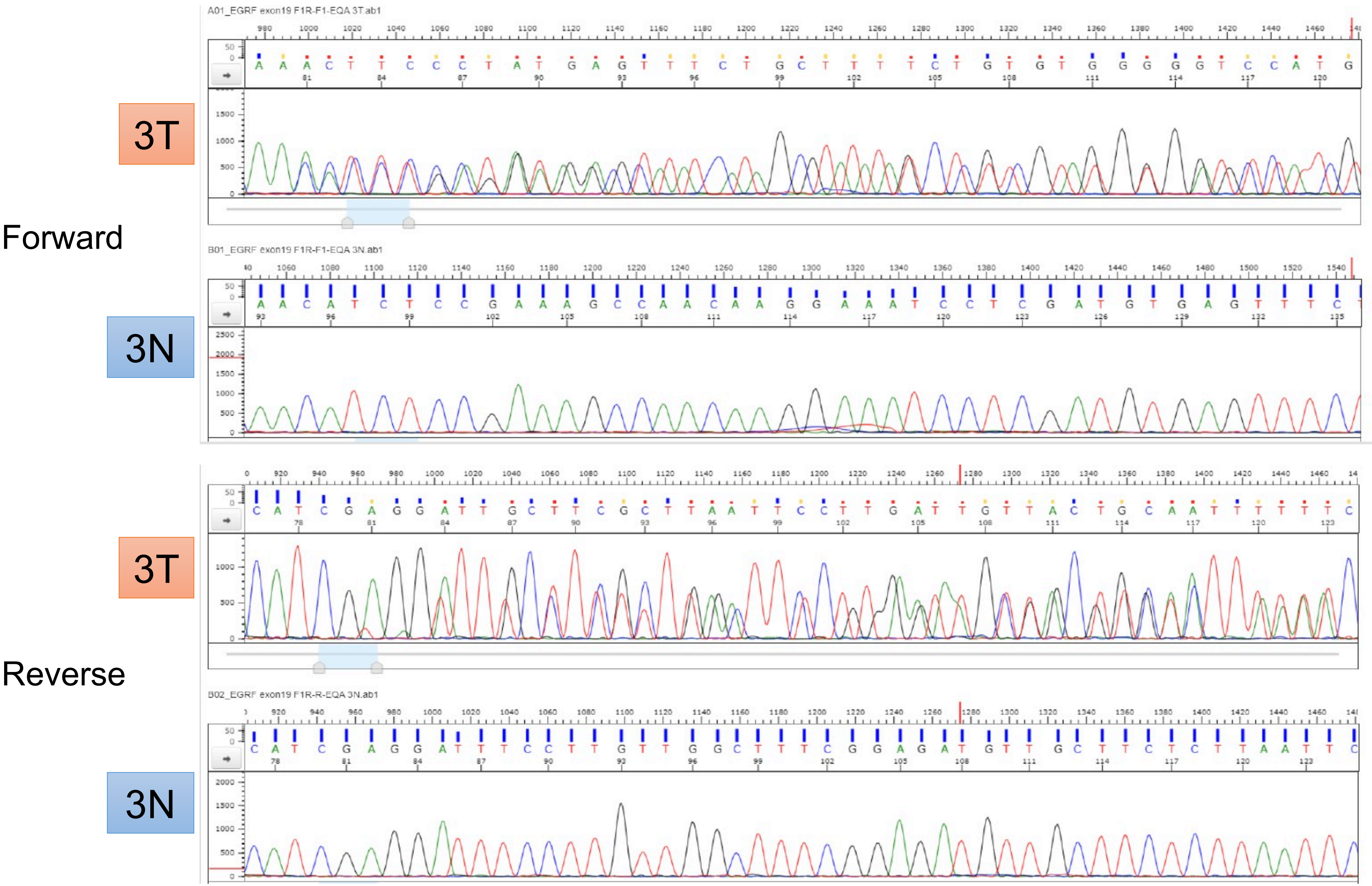


Supplementary Fig. 4

**Patient 4**
Ascending colon cancer
Tumor density=25%

(a) (b)

| (a) | No. of laboratories | | | |
|---|---|---|---|---|
| | Report | | | out of reportable range |
| | 1st time | 2nd time | not detected | |
| ***BRAF* p.Val600Glu** | 8 | 1 | 1 | 0 |
| *APC* p.Thr1556Asn fsTer3 | 5 | 1 | 0 | 4 |
| *APC* p.Asp849Ile fsTer12 | 4 | 0 | 1 | 5 |
| *FBXW7* p.Arg465Cys | 7 | 0 | 0 | 3 |
| *PALB2* p.Phe612Ser fsTer4 | 5 | 0 | 1 | 4 |
| *ALK* p.Ala1300Val | 5 | 1 | 0 | 4 |
| *PDGFRA* p.Ala401Val | 5 | 0 | 1 | 4 |
| *TSC1* p.Glu860Lys | 5 | 1 | 0 | 4 |
| *BRCA2* p.Asp364Asn | 5 | 1 | 0 | 4 |
| *JAK1* p.Lys860Asn fsTer16 | 4 | 0 | 1 | 5 |
| *CREBBP* p.Asn722Asp | 5 | 1 | 0 | 4 |

(c)

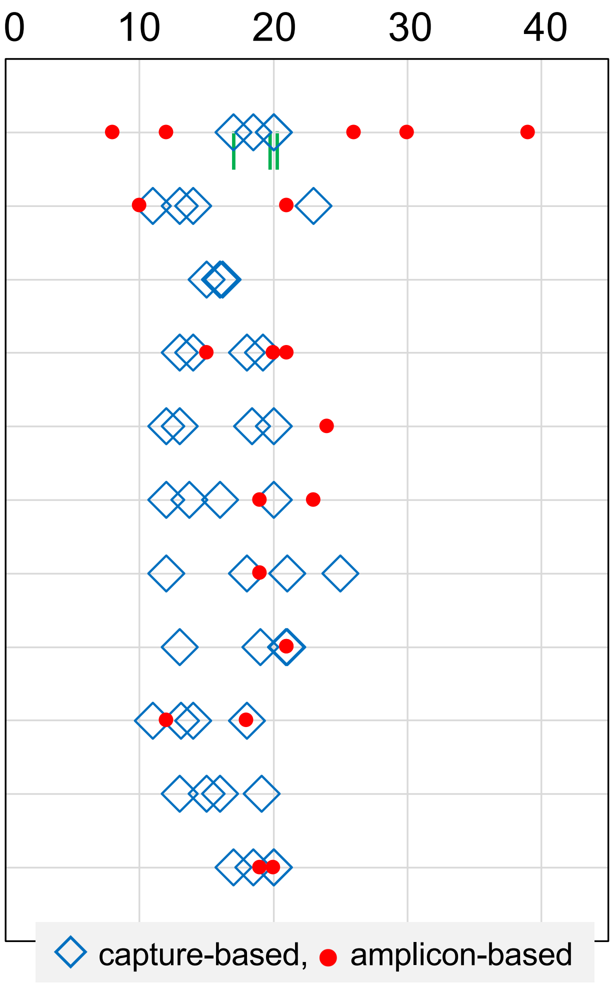


(d)

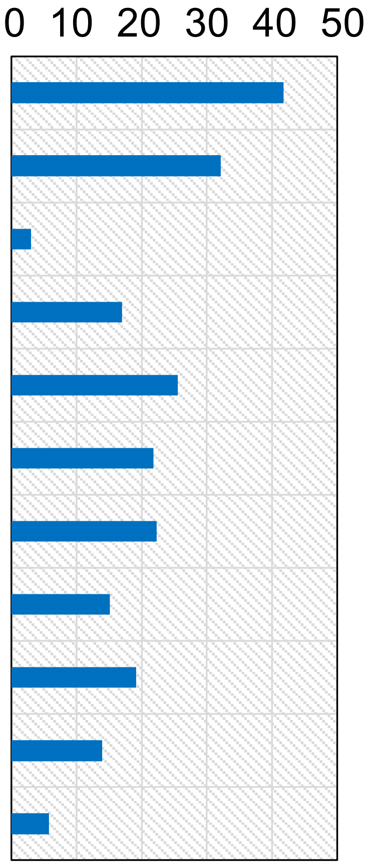


Supplementary Fig. 5

**Patient 5**
Lung cancer
Tumor density=60%

(a)

| Variant |
|---|
| ***EGFR* p.Leu858Arg c.2573T>G** |
| *EGFR* p.Ala289Val c.866C>T |
| *TP53* p.Phe134Leu c.402T>G |
| *RET (G)* p.Ser649Leu c.1946C>T |

(b)

| No. of laboratories | | | |
|---|---|---|---|
| Report | | | out of reportable range |
| 1st time | 2nd time | not detected | |
| 15 | 0 | 0 | 0 |
| 15 | 0 | 0 | 0 |
| 10 | 0 | 0 | 5 |
| 8 | 4 | 0 | 3 |

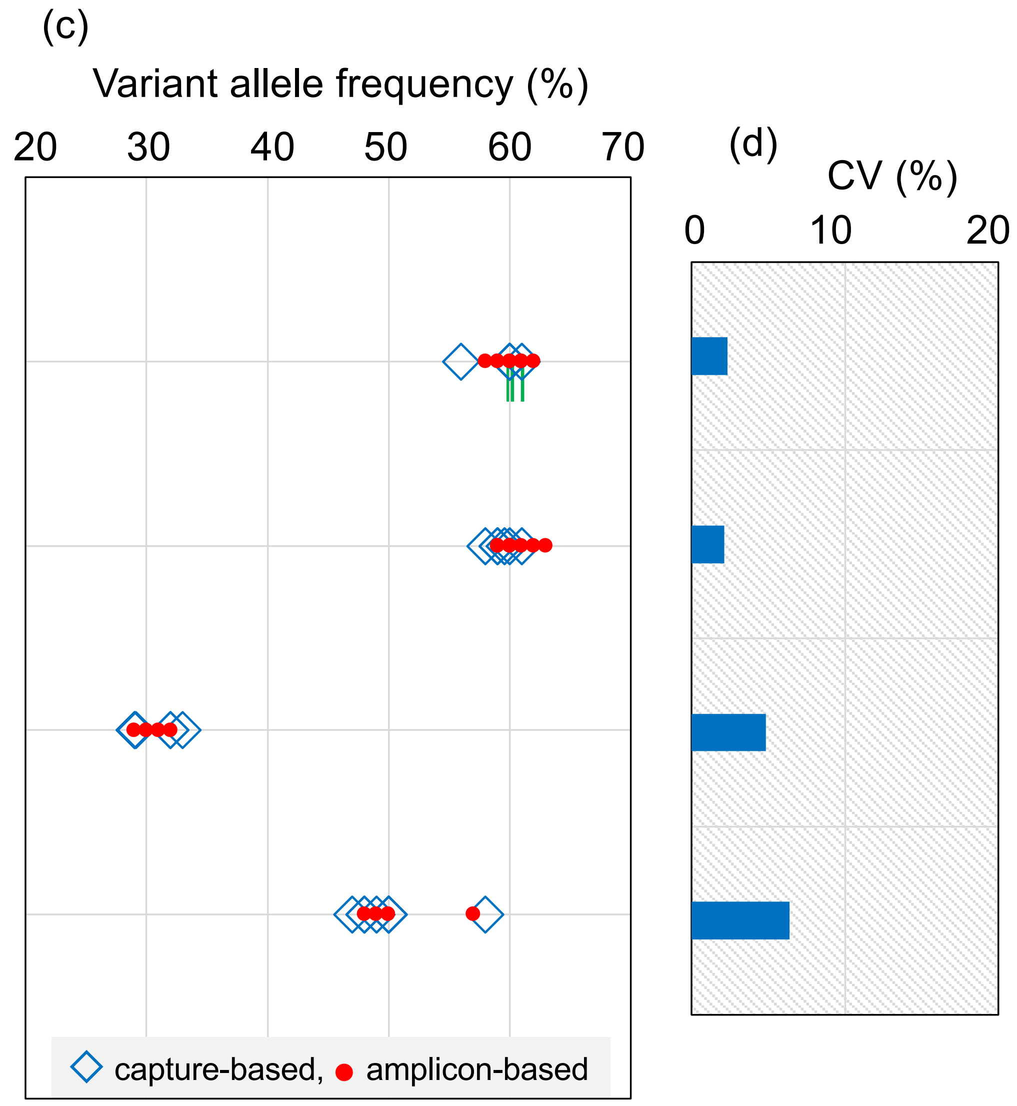


Supplementary Fig. 6

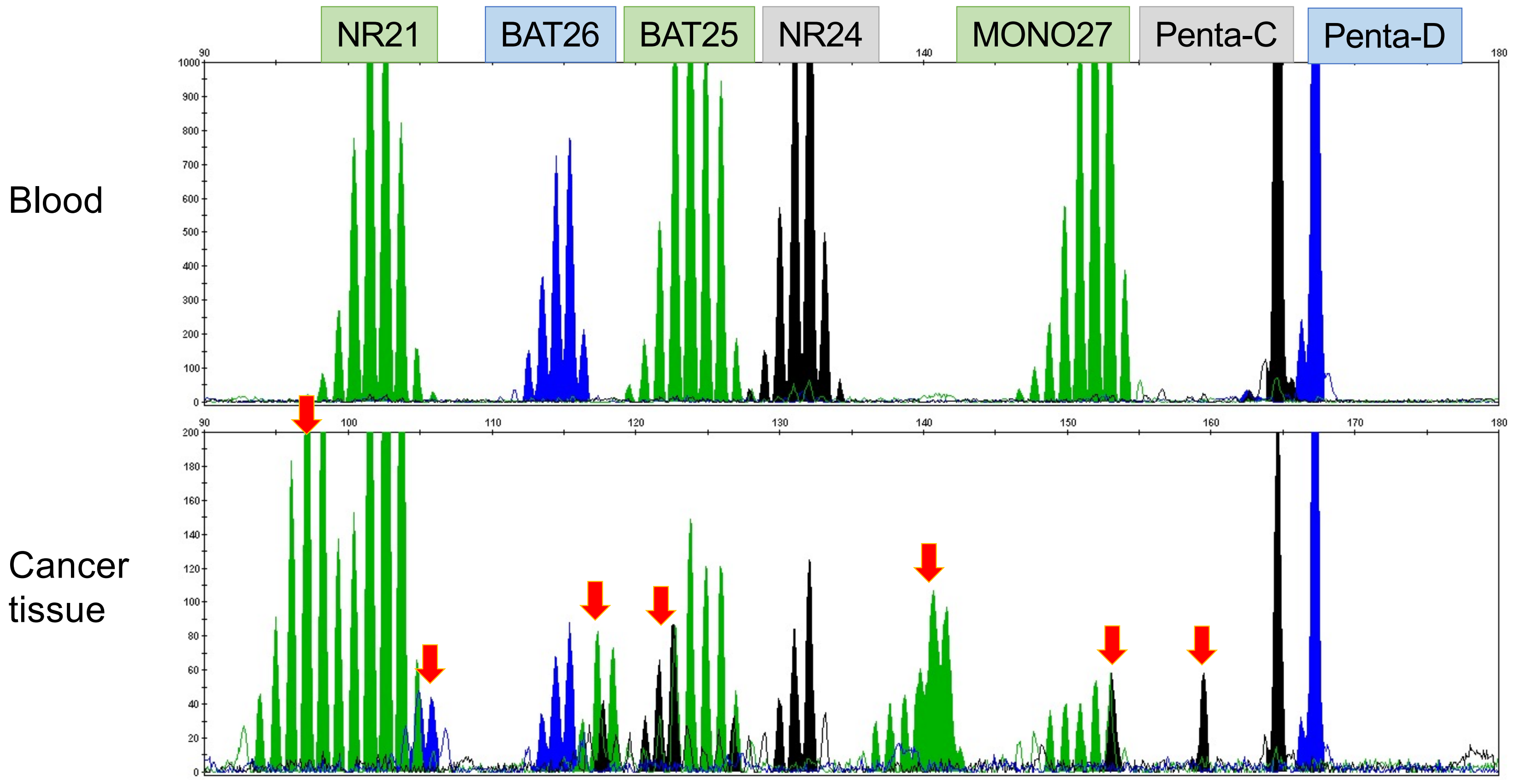


Supplementary Fig. 7

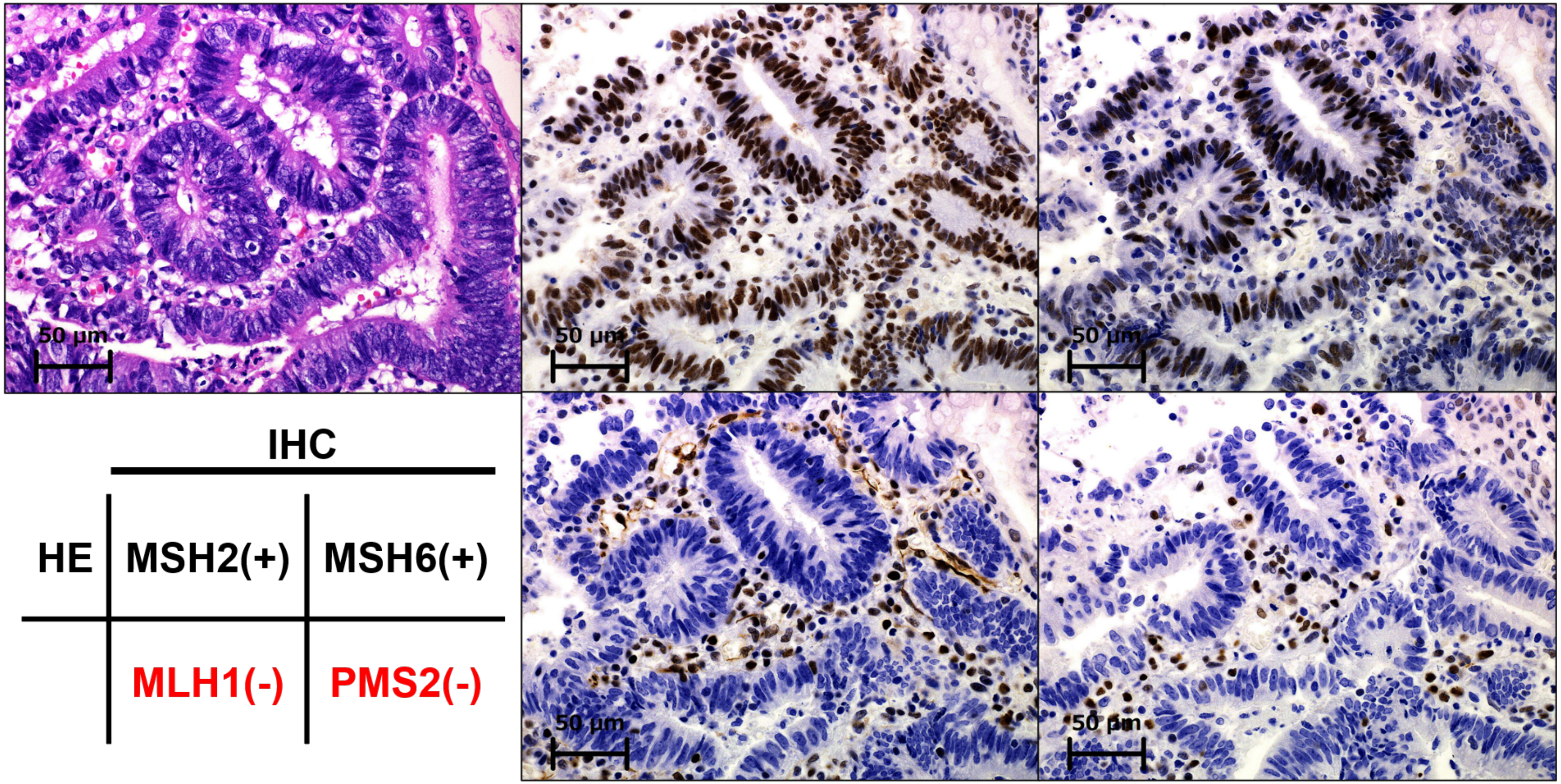


Supplementary Fig. 8

**Supplementary Table 1. Observed variants in the five PT samples**

| Patient No | Cancer Location | Variant | Gene | Position (ch:nt) | HGVSc | HGVSp | VAF% (Range) | COSMIC v92, released 27-AUG-20 | | | dbSNP | ClinVar | | CIViC |
|---|---|---|---|---|---|---|---|---|---|---|---|---|---|---|
| | | | | | | | | Mutation ID | FATHMM prediction | Drug resistance | rsNo. | Accession | Interpretation | Variant Evidence Score |
| 1 | Rectum | missense | *KRAS* | 12:25398281 | NM_033360.2:c.38G>A | NP_203524.1:p.Gly13Asp | 26 - 36 | COSV55497388 | Pathogenic (score 0.98) | Cetuximab,Panitumumab | rs112445441 | VCV000012580.8 | Pathogenic | B - Clinical |
| | | nonsense | *APC* | 5:112154992 | NM_000038.5:c.1263G>A | NP_000029.2:p.Trp421Ter | 32 - 38 | COSV57320574 | Pathogenic (score 0.99) | n/a | – | – | – | |
| | | frameshift | *APC* | 5:112175238 | NM_000038.5:c.3948_3952dupTGAAG | NP_000029.2:p.Asp1318ValfsTer5 | 22 - 34 | – | – | – | – | – | – | |
| | | missense | *PDGFRA* | 4:55151625 | NM_006206.4:c.2411G>A | NP_006197.1:p.Arg804Gln | 28 - 34 | – | – | – | rs571523023 | VCV000407409.3 | Uncertain significance | |
| | | missense | *CTNNB1* | 3:41277224 | NM_001904.3:c.1693C>T | NP_001895.1:p.Arg565Cys | 17 - 28 | – | – | – | – | – | – | |
| | | missense | *BRCA1* (G) | 17:41244526 | NM_007300.3:c.3022A>G | NP_009231.2:p.Met1008Val | 41 - 58 | – | – | – | rs56321129 | VCV000054755.8 | Benign | |
| 2 | Rectum | missense | *KRAS* | 12:25378647 | NM_033360.2:c.351A>C | NP_203524.1:p.Lys117Asn | 45 - 53 | COSV55545304 | Pathogenic (score 0.92) | n/a | rs770248150 | VCV000375965.3 | Pathogenic | |
| | | missense | *KRAS* | 12:25378640 | NM_033360.2:c.358T>A | NP_203524.1:p.Leu120Met | 43 - 52 | – | – | – | – | – | – | |
| | | nonsense | *APC* | 5:112174438 | NM_000038.5:c.3147G>A | NP_000029.2:p.Trp1049Ter | 38 - 54 | – | – | – | rs863225340 | VCV000217964.4 | Pathogenic | |
| | | frameshift | *APC* | 5:112175211 | NM_000038.5:c.3927_3931delAAAGA | NP_000029.2:p.Glu1309AspfsTer4 | 17 - 26 | COSV57321812 | n/a | n/a | rs121913224 | VCV000000816.12 | Pathogenic | |
| | | frameshift | *MYC* | 8:128753087 | NM_002467.4:c.1249dupT | NP_002458.2:p.Tyr417LeufsTer15 | 21 - 27 | – | – | – | – | – | – | |
| | | missense | *CTNNB1* | 3:41267225 | NM_001904.3:c.809A>C | NP_001895.1:p.Lys270Thr | 36 - 42 | – | – | – | – | – | – | |
| 3 | Lung | inframe deletion | *EGFR* | 7:55242479 | NM_005228.3:c.2252_2275delCATCTCCGAAAGCCAACAAGGAAA | NP_005219.2:p.Thr751_Glu758del | 17 - 50 | COSV51795094 | n/a | n/a | – | – | – | |
| | | missense | *EGFR* | 7:55242506 | NM_005228.3:c.2276T>A | NP_005219.2:p.Ile759Asn | 22 - 50 | COSV51795112 | Pathogenic (score 0.99) | n/a | – | – | – | |
| | | frameshift | *TP53* | 17:7579446 | NM_000546.5:c.239_240delCT | NP_000537.3:p.Pro80Hisfs Ter68 | 52 - 62 | – | – | – | – | – | – | |
| | | missense | *FGFR3* | 4:1806575 | NM_001163213.1:c.1297A>G | NP_001156685.1:p.Met433Val | 22 - 27 | – | – | – | – | – | – | |
| | | missense | *STK11* (G) | 19:1221319 | NM_000455.4:c.842C>T | NP_000446.1:p.Pro281Leu | 26 - 46 | COSV58821563 | Pathogenic (score 0.81) | n/a | rs121913322 | VCV000142115.9 | Conflicting interpretations of pathogenicity | |
| 4 | Colon | missense | *BRAF* | 7:140453136 | NM_004333.4:c.1799T>A | NP_004324.2:p.Val600Glu | 8 - 39 | COSV56056643 | Pathogenic (score 0.99) | Cetuximab,Imatinib | rs113488022 | VCV000013961.13 | Pathogenic, drug response | A - Validated |
| | | frameshift | *APC* | 5:112175951 | NM_000038.5:c.4666dupA | NP_000029.2:p.Thr1556AsnfsTer3 | 10 - 23 | COSV57320557 | n/a | n/a | – | – | – | |
| | | frameshift | *APC* | 5:112173831 | NM_000038.5:c.2544delA | NP_000029.2:p.Asp849IlefsTer12 | 15 - 16 | COSV57341877 | n/a | n/a | – | – | – | |
| | | missense | *FBXW7* | 4:153249385 | NM_033632.3:c.1393C>T | NP_361014.1:p.Arg465Cys | 13 - 20 | COSV55891008 | Pathogenic (score 0.80) | n/a | rs867384286 | VCV000376414.1 | Likely pathogenic | |
| | | frameshift | *PALB2* | 16:23641639 | NM_024675.3:c.1835_1836delTT | NP_078951.2:p.Phe612SerfsTer4 | 12 - 24 | – | – | – | – | – | – | |
| | | missense | *ALK* | 2:29430076 | NM_004304.4:c.3899C>T | NP_004295.2:p.Ala1300Val | 12 - 20 | – | – | – | – | – | – | |
| | | missense | *PDGFRA* | 4:55136880 | NM_006206.4:c.1202C>T | NP_006197.1:p.Ala401Val | 12 - 25 | – | – | – | – | – | – | |
| | | missense | *TSC1* | 9:135776149 | NM_000368.4:c.2578G>A | NP_000359.1:p.Glu860Lys | 13 - 21 | – | – | – | – | – | – | |
| | | missense | *BRCA2* | 13:32906705 | NM_000059.3:c.1090G>A | NP_000050.2:p.Asp364Asn | 11 - 18 | – | – | – | – | – | – | |
| | | frameshift | *JAK1* | 1:65306997 | NM_002227.2:c.2580delA | NP_002218.2:p.Lys860AsnfsTer16 | 13 - 19 | COSV61086989 | n/a | n/a | – | – | – | |
| | | missense | *CREBBP* | 16:3824689 | NM_004380.2:c.2164A>G | NP_004371.2:p.Asn722Asp | 17 - 20 | – | – | – | – | – | – | |
| 5 | Lung | missense | *EGFR* | 7:55259515 | NM_005228.3:c.2573T>G | NP_005219.2:p.Leu858Arg | 56 - 62 | COSV51765161 | Pathogenic (score 0.98) | n/a | rs121434568 | VCV000016609.4 | drug response | A - Validated |
| | | missense | *EGFR* | 7:55221822 | NM_005228.3:c.866C>T | NP_005219.2:p.Ala289Val | 59 - 63 | COSV51765841 | Pathogenic (score 0.99) | n/a | rs149840192 | VCV000376209.1 | Likely pathogenic | |
| | | missense | *TP53* | 17:7578528 | NM_000546.5:c.402T>G | NP_000537.3:p.Phe134Leu | 29 - 33 | COSV52814502 | Pathogenic (score 0.95) | n/a | – | – | – | |
| | | missense | *RET* (G) | 10:43609994 | NM_020975.4:c.1946C>T | NP_066124.1:p.Ser649Leu | 47 - 58 | COSV60687010 | Pathogenic (score 0.95) | n/a | rs148935214 | VCV000024928.13 | Conflicting interpretations of pathogenicity | |

*(G)* means germ line mutation.

Supplementary Table 2. Patients' clinical and pathological features

| Patient | Age | Gender | Chief complaint | Location | Pathological findings |
|---|---|---|---|---|---|
| 1 | 70s | Male | Hematochezia | Rectum | Adenocarcinoma, tub2>tub1>muc<br>Ra, 2/5-circ, 57 x 45 mm, Type 2, pT3(SS), INFb, Ly0(D2-40), V1c(SS)(EVG), BD1, Pn0, pN0[0/8], pPM0, pDM0, pRM0(0.5 mm); pT3 pN0 M0 pStage IIa (JSCCR 9th)/pT3 pN0 M0 pStage IIA(UICC 8th) |
| 2 | 60s | Female | Constipation | Rectum | Adenocarcinoma, tub2<br>RS-Ra, circ, 85 x 55 mm, Type 2, pT4a(SE), INFb, Ly1b(MP)(D2-40), V1c(SS)(EVG), Pn1a, BD2, pN1b[2/25], pPM0, pDM0, pRM0; pT4a pN1b M0 pStage IIIb (JSCCR 9th)/pT3 pN0 M0 pStage IIIB(UICC 8th) |
| 3 | 70s | Female | Chest abnormal opacity | Lung | Invasive adenocarcinoma (papillary adenocarcinoma with partial colloid adenocarcinoma-like appearance; papillary: 50%, lepidic: 45%, micropapillary/acinar: 5%)<br>RU, total size: 32 x 19 x 14 mm + 22 x 19 x 18 mm, invasive size: 35 x 16 x 11 mm, pT2a, G2>G1, pm0, pl0, Ly0(D2-40), V0(EVG), STAS(few+), pN0[0/32], br(-); pT2a pN0 M0 pStage IB (JLCS 8th/UICC 8th) |
| 4 | 60s | Female | Fecal occult blood | Ascending colon | Adenocarcinoma, tub1>tub2>muc<br>A, 4/5-circ, 53 x 42 mm, pType 2, pT3(SS/A), INFb, Ly0(D2-40), V1a(EVG), Pn0, BD1, pN0[0/22], pPM0, pDM0, pRM0; pT3 pN0 M0 pStage IIa (JSCCR 9th)/pT3 pN0 M0 pStage IIA (UICC 8th) |
| 5 | 70s | Female | Chest abnormal opacity | Lung | Invasive adenocarcinoma (acinar: 40%, papillary: 30%, solid: 30%)<br>RL, total and invasive size: 17 x 17 x 15 mm, pT1b, G2-3 pl0, pm0, Ly0(D2-40), V1(EVG), STAS(+), pN0[0/12] pa(-), pv(-), br(-); pT1b pN0 M0 pStage IA2 (JLCS 8th/UICC 8th) |

Pathological findigs were followed by the Japanese termes.

JSCCR: Japanese Society for Cancer of the Colon and Rectum (2019) Japanese Classification of Colorectal, Appendiceal, and Anal Carcinoma: the 3d English edition [secondary publication]. J Anus Rectum Colon 3(4):175–195. https://doi.org/10.23922/jarc.2019-018

JLCS: The Japanese Lung Cancer Society (2017) General Rule for Clinical and Pathological Record of Lung Cancer: the 8th edition.  Kanehara & Co., Ltd (in Japanese)

**Supplementary Table 3. Brief summary of the analysis platform and bioinformatics pipeline of the participated laboratories**

| Laboratory ID | a | b | c | d | e | f | g | h | i | j | k | l | m | n | o | a |
|---|---|---|---|---|---|---|---|---|---|---|---|---|---|---|---|---|
| Subject (N/T) | **N/T matched pair** | **N/T matched pair** | **N/T matched pair** | **N/T matched pair** | **N/T matched pair** | Tumor only | Tumor only | **N/T matched pair** | **N/T matched pair** | Tumor only | Tumor only | Tumor only | Tumor only | Tumor only | Tumor only | **N/T matched pair** |
| Library preparation | Hybridization based target capture | | | | | | Multiplex PCR based (Amplicon) | | | | | | | | | |
| Gene panel | Custom panel (SureSelect XT HS) | NCC oncopanel (SureSelect XT HS) | OncoGuide NCC oncopanel (SureSelect XT HS) | Custom panel (SureSelect XT HS) | Custom panel (SureSelect XT HS) | OncoPrime (SureSelect) | GeneRead DNAseq Comprehensive Cancer Panel V2 (QIAGEN) | GeneReadComprehensive Cancer Panel (QIAGEN) | Ion AmpliSeq Cancer Hotspot Panel (Thermo) | Oncomine™FocusAssay （Thermo） | Oncomine™FocusAssay （Thermo） | Oncomine Comprehensive Assay v3 | Oncomine Dx Target Test multi CDx system (Thermo) | Oncomine Dx Target Test multi CDx system (Thermo) | Oncomine Dx Target Test multi CDx system (Thermo) | QIAseq Targeted DNA Panel DHS-002Z+ (QIAGEN) |
| Target gene number | 119 | 114 | 114 | 114+α | 151 | 223 | 160 | 160 | 50<br>(Hotspot genes ) | 52 | 52 | 161 | 46 | 46 | 46 | 72 |
| Analyzed patient Nos. | 1, 2, 3, 4, 5 | 1, 2, 3, 4, 5 | 1, 2, 3, 4, 5 | 1, 2, 3, 4, 5 | 5 | 2, 5 | 1, 2, 3, 4, 5 | 2, 5 | 1, 2, 3, 4, 5 | 1, 2, 3, 4, 5 | 2, 5 | 1, 2, 3, 4, 5 | 1, 2, 3, 4, 5 | 3, 5 | 1, 2, 3, 4, 5 | 2 |

Bioinformatics pipeline

| | | | | | | | | | | | | | | | | |
|---|---|---|---|---|---|---|---|---|---|---|---|---|---|---|---|---|
| Mutation exclusion criteria | Exon/Splicing,-Syn,-SNP(+COSMIC),<br>VAF>=0.10<br>MAF<0.01 | Exon/Splicing,-Syn,-SNP(+COSMIC),<br>VAF>=0.05 | Exon/Splicing,-Syn,-SNP(+COSMIC),<br>VAF>=0.05 | Exon/Splicing,-Syn,-SNP(+COSMIC),<br>VAF>=0.05 | Exon/Splicing,-Syn,-SNP(+COSMIC),<br>VAF>=0.05 | Exon/Splicing,-Syn,-SNP(+COSMIC),<br>VAF>=0.05 | Exon/Splicing,-Syn,-SNP(+COSMIC),<br>VAF>=0.10 or Variant reads >10<br>MAF<0.01 | Exon/Splicing,-Syn,-SNP(+COSMIC),<br>VAF>=0.05<br>MAF<0.01 | Exon/Splicing,-Syn,-SNP(+COSMIC),<br>VAF>=0.05<br>MAF<0.01 | VAF ( SNP >0.04<br>InDel >0.07<br>Hotspot >0.03<br>MNP >0.04 ) | Exon/Splicing,-Syn,-SNP(+COSMIC),<br>VAF>=0.05<br>Total read depth>=250 | - | - | - | - | Exon/Splicing,-Syn,-SNP(+COSMIC),<br>VAF>=0.05<br>MAF<0.01 |
| Database | refGene ,<br>ensGene,<br>1000 Genomes phase 3,<br>ExAC r0.3.1,<br>HGVD v2.10,<br>COSMIC ,<br>ClinVar | EPDB _v5.2,<br>refGene,<br>ensGene,<br>1000 Genomes phase 3,<br>ESP6500 V2-SSA137,<br>ExAC r0.3.1,<br>HGVD v2.10,<br>COSMIC v71,<br>ClinVar,<br>Except v1.00 | EPDB _v5.2,<br>refGene,<br>ensGene,<br>1000 Genomes phase 3,<br>ESP6500 V2-SSA137,<br>ExAC r0.3.1,<br>HGVD v2.10,<br>COSMIC v71,<br>ClinVar,<br>Except v1.00 | EPDB<br>EPDBforPed_v1_19920,<br>refGene,<br>ensGene,<br>1000 Genomes phase 3,<br>ESP6500 V2-SSA137,<br>ExAC r0.3.1,<br>HGVD v2.10,<br>COSMIC v71,<br>ClinVar,<br>Except v1.00 | EPDB<br>20200303_v6.0,<br>refGene,<br>ensGene,<br>1000 Genomes phase 3,<br>ESP6500 V2-SSA137,<br>ExAC r0.3.1,<br>HGVD v2.10,<br>COSMIC v71,<br>ClinVar,<br>Except v2.00 | OMIM,<br>ClinVar,<br>COSMIC,<br>1000Genome,<br>ExAC,<br>ESP6500 | dbNSFP 3.2c ,<br>COSMIC 81 ,<br>ExAC 1 ,<br>dbSNP b153 ,<br>ClinVar 2019-09<br>,<br>HGVD 2.3 ,<br>ToMMo 2kJPN ,<br>CIViC ,<br>GENCODE 31 | COSMIC (84),<br>Clinvar,<br>dpSNP,<br>dbNSFP,<br>gnomAD (2.1),<br>1000 Genomes Project (Phase 3 v5a),<br>OMIM | dra,<br>drugbank,<br>go,<br>hg19_clinvar,<br>hg19_cosmic_85,<br>hg19_dbsnp_151,<br>hg19_dgv,<br>hg19_esp6500,<br>hg19_exac_1,<br>hg19_pfam_31,<br>hg19_phylop,<br>hg19_refgene_89,<br>namedVariants_1,<br>omim,<br>canonical_refseq_hg19_v89 | 5000Exomes,<br>Canonical RefSeq Transcripts v95,<br>ClinVar ,<br>dbSNP 153,<br>DGV,<br>DrugBank ,<br>ExAC (v1),<br>Gene Ontology,<br>Pfam 32,<br>PhyloP Scores,<br>RefSeq Gene Functional Canonical Transcripts Scores 10,<br>RefSeq GeneModel 95,<br>US-FDA labels<br>US-NCCN guidelines<br>EMA labels<br>ESMO guidelines<br>Global clinical trials | 5000Exomes ,<br>Canonical RefSeq Transcripts (v89),<br>ClinVar,<br>dbSNP (v151),<br>DGV ,<br>DrugBank,<br>ExAC (v1),<br>Gene Ontology ,<br>Pfam (v31),<br>PhyloP Scores,<br>RefSeq Functional Canonical Transcripts Scores (v8),<br>RefSeq GeneModel (v89),<br>Named Variants (v1) | dbSNP,<br>ClinVar,<br>COSMIC,<br>VariantDB,<br>ExAC | - | COSMIC v74 | - | refGene ,<br>ensGene,<br>1000 Genomes phase 3,<br>ExAC r0.3.1,<br>HGVD v2.10,<br>COSMIC ,<br>ClinVar |
